# (*OC*-6-35)-(2,2′-Bipyridine-κ^2^ *N*,*N*′)dimeth­yl(3-sulfido­propionato-κ^2^ *S*,*O*)platinum(IV)

**DOI:** 10.1107/S1600536811013626

**Published:** 2011-04-16

**Authors:** Matthew S. McCready, Richard J. Puddephatt

**Affiliations:** a Department of Chemistry, University of Western Ontario, London, Canada N6A 5B7

## Abstract

The title complex, [Pt(CH_3_)_2_(SCH_2_CH_2_CO_2_)(C_10_H_8_N_2_)], is formed by the unusual oxidative addition of the disulfide, *R*_2_S_2_ (*R* = CH_2_CH_2_CO_2_H), to (2,2′-bipyridine)­dimethyl­platin­um(II) with elimination of *R*SH. The product contains an unusual six-membered thiol­ate–carboxyl­ate chelate ring. This slightly distorted octa­hedral complex exhibits *cis* angles ranging from 77.55 (11) to 97.30 (8)° due to the presence of the thiol­ate–carboxyl­ate chelate ring and the constrained bipyridine group. The crystal packing appears to be controlled by a combination of π-stacking [centroid–centroid distance = 3.611 (2) Å] and C—H⋯O inter­actions.

## Related literature

For general background to metal complexes with thiol­ate–carboxyl­ate chelates, see: Henderson *et al.* (2000 ▶); McCready & Puddephatt (2011 ▶); Phillips & Burford (2008 ▶). For the utility and application of disulfides and their reactivity towards transition metals, see: Aye *et al.* (1993 ▶); Bonnington *et al.* (2008 ▶); Wei *et al.* (2005 ▶). For normal ranges of bond angles at platinum(IV) between *cis* ligands, see: Achar *et al.* (1993 ▶); Aye *et al.* (1988 ▶). For inter­planar spacing between bipyridine rings in platinum(IV) complexes of 2,2′-bipyridine, see: Au *et al.* (2009 ▶). For the preparation of dimeth­yl(2,2′-bipyridine)­plat­inum(II), see: Monaghan & Puddephatt (1984 ▶).
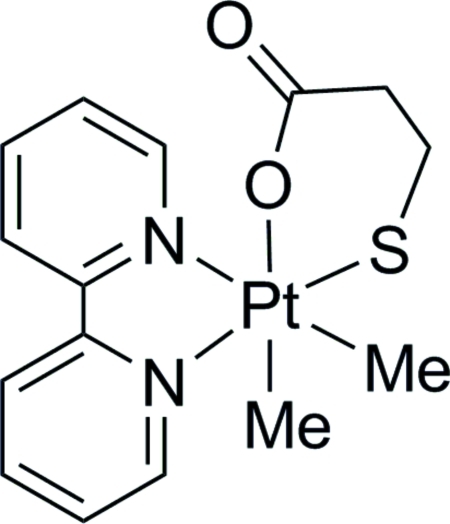


## Experimental

### 

#### Crystal data

[Pt(CH_3_)_2_(C_3_H_4_O_2_S)(C_10_H_8_N_2_)]
*M* *_r_* = 485.46
Monoclinic, 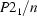

*a* = 14.0759 (6) Å
*b* = 7.7487 (3) Å
*c* = 14.2306 (5) Å
β = 98.978 (2)°
*V* = 1533.11 (10) Å^3^
*Z* = 4
Mo *K*α radiation
μ = 9.29 mm^−1^
*T* = 150 K
0.04 × 0.04 × 0.02 mm

#### Data collection

Bruker APEXII CCD diffractometer
Absorption correction: multi-scan (*SADABS*; Bruker, 2006 ▶) *T*_min_ = 0.708, *T*_max_ = 0.858
52534 measured reflections
4672 independent reflections
3735 reflections with *I* > 2σ(*I*)
*R*_int_ = 0.074

#### Refinement

*R*[*F* ^2^ > 2σ(*F* ^2^)] = 0.026
*wR*(*F* ^2^) = 0.049
*S* = 1.04
4672 reflections
192 parameters
H-atom parameters constrained
Δρ_max_ = 0.92 e Å^−3^
Δρ_min_ = −1.42 e Å^−3^

### 

Data collection: *APEX2* (Bruker, 2007 ▶); cell refinement: *SAINT* (Bruker, 2007 ▶); data reduction: *SAINT*; program(s) used to solve structure: *SHELXS97* (Sheldrick, 2008 ▶); program(s) used to refine structure: *SHELXL97* (Sheldrick, 2008 ▶); molecular graphics: *SHELXTL* (Sheldrick, 2008 ▶); software used to prepare material for publication: *SHELXTL*.

## Supplementary Material

Crystal structure: contains datablocks global, I. DOI: 10.1107/S1600536811013626/tk2735sup1.cif

Structure factors: contains datablocks I. DOI: 10.1107/S1600536811013626/tk2735Isup2.hkl

Additional supplementary materials:  crystallographic information; 3D view; checkCIF report

## Figures and Tables

**Table:** 

Pt1—C15	2.046 (4)
Pt1—C14	2.048 (4)
Pt1—N1	2.107 (3)
Pt1—O2	2.143 (3)
Pt1—N2	2.149 (3)
Pt1—S1	2.2916 (9)

**Table:** 

C15—Pt1—C14	87.88 (17)
C15—Pt1—N1	96.54 (14)
C14—Pt1—N1	90.17 (13)
C15—Pt1—O2	92.60 (15)
N1—Pt1—O2	86.39 (11)
C14—Pt1—N2	92.92 (14)
N1—Pt1—N2	77.55 (11)
O2—Pt1—N2	86.26 (11)
C15—Pt1—S1	88.64 (12)
C14—Pt1—S1	87.34 (11)
N1—Pt1—S1	174.16 (9)
O2—Pt1—S1	96.09 (7)
N2—Pt1—S1	97.30 (8)

**Table 2:** Hydrogen-bond geometry (Å, °)

*D*—H⋯*A*	*D*—H	H⋯*A*	*D*⋯*A*	*D*—H⋯*A*
C10—H10⋯O2^i^	0.95	2.33	3.175 (5)	148

Symmetry code: (i) 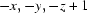.

**Fig. 1.**
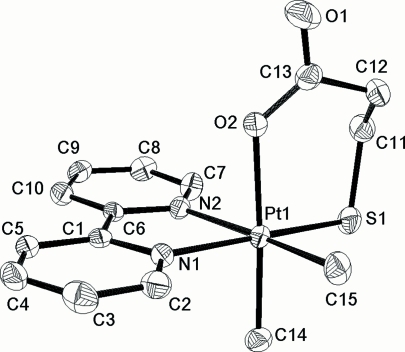
A view of the molecular structure of (I), showing the atom-numbering scheme. Displacement ellipsoids are drawn at the 50% probability level and hydrogen atoms have been omitted.

**Fig. 2.**
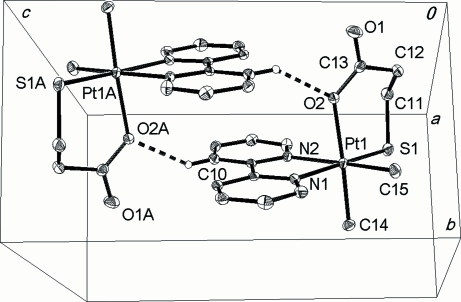
A dimeric unit in (I) showing its orientation within the unit cell. The molecules are related by a centre of inversion and A = -x, 1 - y, -z+1.

## supplementary crystallographic information

### Comment

Activation of the S—S bond of a disulfide, *R*_2_S_2_, has shown significant promise in the areas of medicinal chemistry and catalysis when cleaved by a transition metal complex (Wei *et al.*, 2005). One route of interest for the activation of an S—S bond is the oxidative addition to a transition metal complex, of which there have been many studies involving platinum(II) complexes (Aye *et al.*, 1993; Bonnington *et al.*, 2008). Unexpectedly, the oxidative addition of 3,3'-dithiodipropionic acid led to the formation of a thiolate-carboxylate chelate ring. This type of chelate ring is not without precedent (Henderson *et al.*, 2000; Phillips & Burford, 2008) but the title complex, (I), is the first example of a thiolate-carboxylate chelate with platinum(IV) (McCready & Puddephatt, 2011). In this context, the crystal structure of (I) is presented.

The stereochemistry at the platinum(IV) center is octahedral with two mutually *cis* methyl groups and chelating 2,2'-bipyridine and 3-thiolatopropionato groups (Fig. 1 and Table 1). The distance Pt—N2 = 2.149 (3) Å is significantly longer than Pt—N1 = 2.107 (3) Å, because the methyl group has a higher *trans* influence than the thiolato group. The angles at platinum(IV) between *cis* ligands range from 77.55 (11) to 97.30 (8) °, Table 1, as a result of constraints of the chelate ligands and lie in the expected ranges (Aye *et al.*, 1988; Achar *et al.*, 1993).

The chief intermolecular interactions arise through π-stacking between the bipyridine rings of centrosymmetrically related molecules of (I) (Fig. 2). The mean interplanar spacing between the bipyridine rings comprising the dimer unit is 3.36 Å, which is consistent with observed values of about 3.3 Å for platinum(IV) complexes of 2,2'-bipyridine (Au *et al.*, 2009). The ring centroid(N3-pyridyl)···ring centroid(N4-pyridyl)^A^ distance = 3.611 (2) Å for A = -x, -y, 1-z. The dimer formation through π-stacking is further stabilized by the presence of a weak C10—H···O2 interaction (Table 2).

### Experimental

Dimethyl(2,2'-bipyridine)platinum(II) was prepared according to a previously published procedure by Monaghan and Puddephatt (1984). Spectroscopic Analysis, IR (ν, cm^-1^, KBr disk): ν(Aromatic CH) = 3107, ν(aliphatic CH) = 2847 and 2781; ν(CC) = 1601, 1465 and 1443. ^1^H NMR in acetone-d^6^: δ = 0.97 [s, 6H, ^2^J(PtH) = 86 Hz, MePt); 7.70 [dd, 2H, ^3^J(H_5_H_6_) = 6 Hz, ^3^J(H_5_H_4_) = 7 Hz, H_5_]; 8.33 [dd, 2H, ^3^J(H_4_H_5_) = 7 Hz, ^3^J(H_4_H_3_) = 8 Hz, H_4_]; 8.43 [d, 2H, ^3^J(H_3_H_4_) = 8 Hz, H_3_]; 9.23 [d, 2H, ^3^J(H_6_H_5_) = 6 Hz, H_5_]. A solution of PtMe_2_(bipy) (0.010 g, 0.026 mmol) in minimum acetone was added to a solution of dithiopropionic acid (0.0056 g, 0.026 mmol) in minimum acetone. Mixing of the two solutions led to the formation of a red-brown colour which persists while precipitation of the product is observed. After 30 h the red-orange precipitate can be isolated by decantation of the solvent and was washed with diethyl ether. PtMe_2_(bipy) (0.0010 g) was then dissolved in minimal chlorobenzene and added to an NMR analysis tube. To this solution, a buffer layer of chlorobenzene followed by acetone was added in order to slow the rate of diffusion of the subsequently added acetone solution of (0.0006 g) of dithiopropionic acid. The sample was placed in a cool dark place and allowed to crystallize over the course of two weeks producing crystals of (I). Yields of 68–73% were achieved. Spectroscopic Analysis, IR (ν, cm^-1^, Bruker Tenser 27 FTIR spectrophotometer as KBr disk): ν(Aromatic CH) = 3107; ν(aliphatic CH) = 2847 and 2781; ν(CC) = 1601, 1465 and 1443; ν(CO) = 1710. ^1^H NMR ( acetone-d^6^, p.p.m., Mercury 400 MHz NMR spectrometer): δ = 0.50 [s, 3H, ^2^J(PtH)=74 Hz, MePt *trans* to O]; 1.63 [s, 3H, ^2^J(PtH) = 69 Hz, MePt *trans* to N]; 2.73 [t, 2H, ^3^J(HH) = 7 Hz, CH_2_]; 2.96 [t, 2H, ^3^J(HH)=7 Hz, CH_2_]; 7.42 – 9.78 [8H, aromatic protons (bipy)]. MALDI-MS (CHCA): m/*z* = 499 (PtMe_3_(NN)(S(CH_2_)_2_COO)]^+^, m/*z* = 486 (Complex 1 + H^+^); m/*z* = 381([PtMe_2_(bipy)]^+^). The

### Refinement

The C-bound H atoms were placed in calculated positions (C—H 0.95–0.99 Å) and were included in the refinement in the riding model approximation with *U*_iso_(H) values equal to 1.2*U*_eq_(C) The maximum and minimum residual electron density peaks of 0.92 and -1.42 e Å^-3^, respectively, were located 0.64 Å and 0.74 Å from the Pt1 atom respectively.

### Crystal data

**Table:**

[Pt(CH_3_)_2_(C_3_H_4_O_2_S)(C_10_H_8_N_2_)]	*F*(000) = 928
*M**_r_* = 485.46	*D*_x_ = 2.103 Mg m^−^^3^
Monoclinic, *P*2_1_/*n*	Mo *K*α radiation, λ = 0.71073 Å
Hall symbol: -P 2yn	Cell parameters from 7767 reflections
*a* = 14.0759 (6) Å	θ = 2.2–23.9°
*b* = 7.7487 (3) Å	µ = 9.29 mm^−^^1^
*c* = 14.2306 (5) Å	*T* = 150 K
β = 98.978 (2)°	Block, orange
*V* = 1533.11 (10) Å^3^	0.04 × 0.04 × 0.02 mm
*Z* = 4	

### Data collection

**Table:**

Bruker APEXII CCD diffractometer	4672 independent reflections
Radiation source: fine-focus sealed tube	3735 reflections with *I* > 2σ(*I*)
graphite	*R*_int_ = 0.074
φ and ω scans	θ_max_ = 30.5°, θ_min_ = 1.9°
Absorption correction: multi-scan (*SADABS*; Bruker, 2006)	*h* = −20→20
*T*_min_ = 0.708, *T*_max_ = 0.858	*k* = −11→11
52534 measured reflections	*l* = −20→20

### Refinement

**Table:**

Refinement on *F*^2^	Primary atom site location: structure-invariant direct methods
Least-squares matrix: full	Secondary atom site location: difference Fourier map
*R*[*F*^2^ > 2σ(*F*^2^)] = 0.026	Hydrogen site location: inferred from neighbouring sites
*wR*(*F*^2^) = 0.049	H-atom parameters constrained
*S* = 1.04	*w* = 1/[σ^2^(*F*_o_^2^) + (0.0113*P*)^2^ + 3.0575*P*] where *P* = (*F*_o_^2^ + 2*F*_c_^2^)/3
4672 reflections	(Δ/σ)_max_ = 0.001
192 parameters	Δρ_max_ = 0.92 e Å^−^^3^
0 restraints	Δρ_min_ = −1.42 e Å^−^^3^

### Special details

**Table:**

Geometry. All e.s.d.'s (except the e.s.d. in the dihedral angle between two l.s. planes) are estimated using the full covariance matrix. The cell e.s.d.'s are taken into account individually in the estimation of e.s.d.'s in distances, angles and torsion angles; correlations between e.s.d.'s in cell parameters are only used when they are defined by crystal symmetry. An approximate (isotropic) treatment of cell e.s.d.'s is used for estimating e.s.d.'s involving l.s. planes.
Refinement. Refinement of *F*^2^ against ALL reflections. The weighted *R*-factor *wR* and goodness of fit *S* are based on *F*^2^, conventional *R*-factors *R* are based on *F*, with *F* set to zero for negative *F*^2^. The threshold expression of *F*^2^ > σ(*F*^2^) is used only for calculating *R*-factors(gt) *etc*. and is not relevant to the choice of reflections for refinement. *R*-factors based on *F*^2^ are statistically about twice as large as those based on *F*, and *R*- factors based on ALL data will be even larger.

### Fractional atomic coordinates and isotropic or equivalent isotropic displacement parameters (Å^2^)

**Table:**

	*x*	*y*	*z*	*U*_iso_*/*U*_eq_	
Pt1	0.032981 (10)	0.174968 (18)	0.254103 (9)	0.01904 (4)	
S1	0.12815 (8)	0.07657 (14)	0.14873 (7)	0.0283 (2)	
O1	−0.1206 (2)	−0.2968 (4)	0.2172 (2)	0.0406 (8)	
N1	−0.0415 (2)	0.2693 (4)	0.3609 (2)	0.0195 (6)	
C1	0.0013 (3)	0.2400 (5)	0.4520 (2)	0.0196 (7)	
O2	−0.0384 (2)	−0.0655 (3)	0.26891 (18)	0.0277 (6)	
N2	0.1270 (2)	0.1055 (4)	0.3823 (2)	0.0195 (6)	
C2	−0.1257 (3)	0.3538 (5)	0.3450 (3)	0.0260 (8)	
H2	−0.1548	0.3754	0.2813	0.031*	
C3	−0.1716 (3)	0.4105 (5)	0.4185 (3)	0.0311 (9)	
H3	−0.2312	0.4701	0.4054	0.037*	
C4	−0.1293 (3)	0.3790 (5)	0.5112 (3)	0.0283 (9)	
H4	−0.1599	0.4159	0.5627	0.034*	
C5	−0.0424 (3)	0.2938 (5)	0.5284 (3)	0.0230 (8)	
H5	−0.0126	0.2718	0.5919	0.028*	
C6	0.0944 (2)	0.1480 (4)	0.4640 (2)	0.0182 (7)	
C7	0.2101 (3)	0.0196 (5)	0.3870 (3)	0.0257 (8)	
H7	0.2328	−0.0094	0.3295	0.031*	
C8	0.2641 (3)	−0.0284 (5)	0.4728 (3)	0.0277 (8)	
H8	0.3227	−0.0898	0.4742	0.033*	
C9	0.2314 (3)	0.0143 (5)	0.5566 (3)	0.0256 (8)	
H9	0.2668	−0.0188	0.6162	0.031*	
C10	0.1464 (3)	0.1060 (5)	0.5524 (3)	0.0242 (8)	
H10	0.1239	0.1397	0.6092	0.029*	
C11	0.1008 (3)	−0.1529 (5)	0.1446 (3)	0.0308 (9)	
H11B	0.1342	−0.2073	0.0959	0.037*	
H11A	0.1273	−0.2044	0.2069	0.037*	
C12	−0.0063 (3)	−0.1973 (5)	0.1222 (3)	0.0304 (9)	
H12B	−0.0372	−0.1179	0.0720	0.036*	
H12A	−0.0129	−0.3159	0.0961	0.036*	
C13	−0.0603 (3)	−0.1864 (5)	0.2073 (3)	0.0260 (8)	
C14	0.0972 (3)	0.4106 (5)	0.2463 (3)	0.0272 (8)	
H14A	0.0999	0.4385	0.1796	0.041*	
H14B	0.1625	0.4071	0.2821	0.041*	
H14C	0.0597	0.4990	0.2735	0.041*	
C15	−0.0667 (3)	0.2486 (6)	0.1408 (3)	0.0296 (9)	
H15A	−0.0815	0.3714	0.1466	0.044*	
H15B	−0.1255	0.1806	0.1400	0.044*	
H15C	−0.0409	0.2291	0.0816	0.044*	

### Atomic displacement parameters (Å^2^)

**Table:**

	*U*^11^	*U*^22^	*U*^33^	*U*^12^	*U*^13^	*U*^23^
Pt1	0.02214 (7)	0.01977 (7)	0.01574 (6)	0.00002 (7)	0.00464 (4)	0.00076 (6)
S1	0.0343 (5)	0.0298 (6)	0.0236 (5)	0.0024 (4)	0.0135 (4)	0.0001 (4)
O1	0.0444 (19)	0.0335 (18)	0.0450 (18)	−0.0164 (15)	0.0105 (15)	−0.0097 (14)
N1	0.0218 (15)	0.0184 (15)	0.0189 (14)	−0.0009 (12)	0.0043 (12)	−0.0014 (12)
C1	0.0235 (18)	0.0144 (17)	0.0219 (17)	−0.0065 (14)	0.0071 (14)	−0.0009 (14)
O2	0.0382 (16)	0.0254 (15)	0.0205 (13)	−0.0109 (12)	0.0081 (12)	−0.0025 (11)
N2	0.0202 (15)	0.0218 (16)	0.0165 (14)	−0.0022 (13)	0.0029 (11)	0.0011 (12)
C2	0.0268 (19)	0.023 (2)	0.0285 (19)	−0.0001 (16)	0.0050 (16)	0.0001 (16)
C3	0.026 (2)	0.028 (2)	0.041 (2)	0.0060 (17)	0.0090 (18)	−0.0030 (18)
C4	0.034 (2)	0.023 (2)	0.032 (2)	−0.0028 (17)	0.0162 (17)	−0.0061 (16)
C5	0.031 (2)	0.019 (2)	0.0215 (17)	−0.0050 (15)	0.0107 (15)	−0.0021 (14)
C6	0.0217 (17)	0.0154 (18)	0.0183 (15)	−0.0055 (14)	0.0054 (13)	−0.0030 (13)
C7	0.0204 (18)	0.034 (2)	0.0231 (18)	0.0031 (16)	0.0038 (14)	0.0014 (16)
C8	0.0207 (18)	0.030 (2)	0.032 (2)	−0.0005 (17)	0.0007 (16)	0.0047 (17)
C9	0.0265 (19)	0.026 (2)	0.0220 (17)	−0.0073 (16)	−0.0028 (15)	0.0045 (15)
C10	0.027 (2)	0.024 (2)	0.0215 (18)	−0.0057 (16)	0.0031 (15)	−0.0022 (15)
C11	0.039 (2)	0.027 (2)	0.0275 (19)	0.0063 (18)	0.0086 (17)	−0.0018 (17)
C12	0.043 (2)	0.025 (2)	0.0225 (18)	0.0005 (19)	0.0037 (17)	−0.0041 (16)
C13	0.0288 (19)	0.024 (2)	0.0237 (17)	0.0000 (18)	−0.0001 (15)	0.0049 (16)
C14	0.032 (2)	0.023 (2)	0.029 (2)	−0.0021 (17)	0.0106 (17)	0.0023 (16)
C15	0.030 (2)	0.033 (2)	0.0233 (19)	0.0091 (18)	−0.0045 (16)	0.0001 (17)

### Geometric parameters (Å, °)

**Table:**

Pt1—C15	2.046 (4)	C5—H5	0.9500
Pt1—C14	2.048 (4)	C6—C10	1.393 (5)
Pt1—N1	2.107 (3)	C7—C8	1.385 (5)
Pt1—O2	2.143 (3)	C7—H7	0.9500
Pt1—N2	2.149 (3)	C8—C9	1.383 (5)
Pt1—S1	2.2916 (9)	C8—H8	0.9500
S1—C11	1.818 (4)	C9—C10	1.384 (5)
O1—C13	1.230 (5)	C9—H9	0.9500
N1—C2	1.342 (5)	C10—H10	0.9500
N1—C1	1.361 (4)	C11—C12	1.531 (6)
C1—C5	1.395 (5)	C11—H11B	0.9900
C1—C6	1.478 (5)	C11—H11A	0.9900
O2—C13	1.287 (5)	C12—C13	1.529 (5)
N2—C7	1.338 (5)	C12—H12B	0.9900
N2—C6	1.355 (4)	C12—H12A	0.9900
C2—C3	1.383 (5)	C14—H14A	0.9800
C2—H2	0.9500	C14—H14B	0.9800
C3—C4	1.382 (6)	C14—H14C	0.9800
C3—H3	0.9500	C15—H15A	0.9800
C4—C5	1.378 (5)	C15—H15B	0.9800
C4—H4	0.9500	C15—H15C	0.9800
			
C15—Pt1—C14	87.88 (17)	C10—C6—C1	123.3 (3)
C15—Pt1—N1	96.54 (14)	N2—C7—C8	122.1 (3)
C14—Pt1—N1	90.17 (13)	N2—C7—H7	118.9
C15—Pt1—O2	92.60 (15)	C8—C7—H7	118.9
C14—Pt1—O2	176.55 (12)	C9—C8—C7	119.0 (4)
N1—Pt1—O2	86.39 (11)	C9—C8—H8	120.5
C15—Pt1—N2	174.03 (14)	C7—C8—H8	120.5
C14—Pt1—N2	92.92 (14)	C8—C9—C10	119.2 (4)
N1—Pt1—N2	77.55 (11)	C8—C9—H9	120.4
O2—Pt1—N2	86.26 (11)	C10—C9—H9	120.4
C15—Pt1—S1	88.64 (12)	C9—C10—C6	119.2 (3)
C14—Pt1—S1	87.34 (11)	C9—C10—H10	120.4
N1—Pt1—S1	174.16 (9)	C6—C10—H10	120.4
O2—Pt1—S1	96.09 (7)	C12—C11—S1	115.0 (3)
N2—Pt1—S1	97.30 (8)	C12—C11—H11B	108.5
C11—S1—Pt1	101.78 (13)	S1—C11—H11B	108.5
C2—N1—C1	119.3 (3)	C12—C11—H11A	108.5
C2—N1—Pt1	125.0 (2)	S1—C11—H11A	108.5
C1—N1—Pt1	115.6 (2)	H11B—C11—H11A	107.5
N1—C1—C5	120.6 (3)	C13—C12—C11	114.7 (3)
N1—C1—C6	116.3 (3)	C13—C12—H12B	108.6
C5—C1—C6	123.0 (3)	C11—C12—H12B	108.6
C13—O2—Pt1	129.2 (2)	C13—C12—H12A	108.6
C7—N2—C6	119.3 (3)	C11—C12—H12A	108.6
C7—N2—Pt1	125.7 (2)	H12B—C12—H12A	107.6
C6—N2—Pt1	114.9 (2)	O1—C13—O2	121.6 (4)
N1—C2—C3	122.2 (4)	O1—C13—C12	119.4 (4)
N1—C2—H2	118.9	O2—C13—C12	119.0 (3)
C3—C2—H2	118.9	Pt1—C14—H14A	109.5
C4—C3—C2	118.9 (4)	Pt1—C14—H14B	109.5
C4—C3—H3	120.6	H14A—C14—H14B	109.5
C2—C3—H3	120.6	Pt1—C14—H14C	109.5
C5—C4—C3	119.5 (3)	H14A—C14—H14C	109.5
C5—C4—H4	120.2	H14B—C14—H14C	109.5
C3—C4—H4	120.2	Pt1—C15—H15A	109.5
C4—C5—C1	119.5 (4)	Pt1—C15—H15B	109.5
C4—C5—H5	120.3	H15A—C15—H15B	109.5
C1—C5—H5	120.3	Pt1—C15—H15C	109.5
N2—C6—C10	121.1 (3)	H15A—C15—H15C	109.5
N2—C6—C1	115.5 (3)	H15B—C15—H15C	109.5
			
C15—Pt1—S1—C11	−98.02 (19)	N1—Pt1—N2—C6	1.7 (2)
C14—Pt1—S1—C11	174.04 (18)	O2—Pt1—N2—C6	−85.4 (2)
N1—Pt1—S1—C11	109.3 (8)	S1—Pt1—N2—C6	178.9 (2)
O2—Pt1—S1—C11	−5.56 (16)	C1—N1—C2—C3	−0.9 (6)
N2—Pt1—S1—C11	81.44 (16)	Pt1—N1—C2—C3	179.1 (3)
C15—Pt1—N1—C2	−2.1 (3)	N1—C2—C3—C4	0.0 (6)
C14—Pt1—N1—C2	85.8 (3)	C2—C3—C4—C5	0.6 (6)
O2—Pt1—N1—C2	−94.3 (3)	C3—C4—C5—C1	−0.2 (6)
N2—Pt1—N1—C2	178.7 (3)	N1—C1—C5—C4	−0.7 (5)
S1—Pt1—N1—C2	150.4 (7)	C6—C1—C5—C4	179.9 (3)
C15—Pt1—N1—C1	177.9 (3)	C7—N2—C6—C10	0.8 (5)
C14—Pt1—N1—C1	−94.2 (3)	Pt1—N2—C6—C10	178.0 (3)
O2—Pt1—N1—C1	85.7 (3)	C7—N2—C6—C1	−179.0 (3)
N2—Pt1—N1—C1	−1.2 (2)	Pt1—N2—C6—C1	−1.9 (4)
S1—Pt1—N1—C1	−29.5 (10)	N1—C1—C6—N2	0.8 (5)
C2—N1—C1—C5	1.3 (5)	C5—C1—C6—N2	−179.8 (3)
Pt1—N1—C1—C5	−178.8 (3)	N1—C1—C6—C10	−179.0 (3)
C2—N1—C1—C6	−179.3 (3)	C5—C1—C6—C10	0.4 (5)
Pt1—N1—C1—C6	0.7 (4)	C6—N2—C7—C8	0.3 (6)
C15—Pt1—O2—C13	56.8 (3)	Pt1—N2—C7—C8	−176.4 (3)
C14—Pt1—O2—C13	155 (2)	N2—C7—C8—C9	−0.3 (6)
N1—Pt1—O2—C13	153.2 (3)	C7—C8—C9—C10	−0.9 (6)
N2—Pt1—O2—C13	−129.1 (3)	C8—C9—C10—C6	2.0 (6)
S1—Pt1—O2—C13	−32.1 (3)	N2—C6—C10—C9	−2.0 (5)
C15—Pt1—N2—C7	170.6 (13)	C1—C6—C10—C9	177.8 (3)
C14—Pt1—N2—C7	−91.9 (3)	Pt1—S1—C11—C12	53.1 (3)
N1—Pt1—N2—C7	178.6 (3)	S1—C11—C12—C13	−81.1 (4)
O2—Pt1—N2—C7	91.5 (3)	Pt1—O2—C13—O1	−162.2 (3)
S1—Pt1—N2—C7	−4.2 (3)	Pt1—O2—C13—C12	20.8 (5)
C15—Pt1—N2—C6	−6.3 (15)	C11—C12—C13—O1	−139.6 (4)
C14—Pt1—N2—C6	91.2 (3)	C11—C12—C13—O2	37.5 (5)

### Hydrogen-bond geometry (Å, °)

**Table:**

*D*—H···*A*	*D*—H	H···*A*	*D*···*A*	*D*—H···*A*
C10—H10···O2^i^	0.95	2.33	3.175 (5)	148

Symmetry codes: (i) −*x*, −*y*, −*z*+1.
